# Use of magnetic fluids in process system for pipe isolations

**DOI:** 10.1016/j.heliyon.2024.e35221

**Published:** 2024-07-26

**Authors:** Jake O. Emmerson, Amirali Shateri, Jianfei Xie

**Affiliations:** a School of Engineering, University of Derby, DE22 3AW, UK; b Musk Process Services, Astron Business Park, DE11 9DW, UK

**Keywords:** Magnetic fluids, Ferrohydrodynamics, Process engineering, Pipe isolation

## Abstract

This paper investigates the use of magnetic fluids known as ferrofluids to act as *ad hoc* valves within pipe systems to create isolation points for stemming pipe leakages and to halt leakages before they become largescale disasters. The sealing abilities of ferrofluids were proven for microvalves (ID ≤ 1 mm) in hydrostatic experiments and extended to the macroscale applications (ID ≥ 6 mm). Theoretical prediction and magnetic finite element analysis (FEA) were also undertaken to predict the burst pressure, and a comparison of both results against the experimental measurement was made. The up-scale results (10–18 mm ID) indicated that it is feasible to develop a ferrofluid that can be extended to approximate the applicable magnet strength to achieve higher burst pressure. It was concluded that the ferrofluid isolation valves hold potential in macroscale environments for process engineering in favour of a positive isolation.

## 1 Introduction

The essence of a fluid piping system is to contain and transport its fluid. However, with equipment breakdowns, gasket failures or physical damage, leakages can occur. As detailed in many forensic engineering cases, even small leakages can escalate to the larger disastrous events [1]. On a domestic level a small water leak may not because for concern. Unfortunately, when leakages transpire at an industrial level with volatile chemicals, catastrophic events such as the 2010 Deepwater Horizon oil spill [2] can occur. When these events happen, the exacerbating factor is the lack of valving or ability to isolate the fluid and stem the flow quickly. Being able to create valves on demand in pipelines will help lead to quicker resolutions, i.e., reducing the scale of these environmental tragedies. Process plants are used for a variety of purposes, such as mass-producing foods and pharmaceuticals to generating hydrogen or oil products. In industries with high value or volatile products, the leak tightness of a system is paramount to its safe operation. One method to reduce this risk is to minimize breaks in pipework, reducing the number of valves, reducing the gasket connections, essentially the less gaskets you have the less chance one fails.

Since the inception of magnetic fluids for rocket fuel in 1960's, ferrofluids have found many successful applications. Within the realm of sealing arrangements, the properties of ferrofluids are very advantageous, such as the high adaptability and practically zero surface friction with the sealing faces [3] (micro-magnetofluids in microfluidic systems) although the durability and success of the seals are highly dependent on the arrangement or environment [4,5]. The capabilities of ferrofluids have been capitalised for sealing around shafts in items such as pumps, motors, and microelectronic devices [6]. For many years since Rosenweig and Ferrotec's original patent in 1970, there have been continuing efforts to optimise and improve the performance of ferrofluid-based shaft seals [7].

Ferrofluids are a manmade dispersion of magnetic nanoparticles in a liquid carrier fluid, whereby the nanoparticles avoid agglomeration through surfactants and thermal agitation [[8], [9], [10]]. In the absence of a magnetic force the particles float freely yet when a magnetic field is applied; ordered chains of nanoparticles are created as the magnetic domains of each particle align. This alignment of chains gives the ferrofluid its strength to resist pressure (i.e., resisting force per unit area), with the surfactant and carrier fluid providing the boundary against the opposing fluids between the particles. Ferrofluids had already been considered for use in valves, with the earliest published mentioning of ferrofluid micro-valving in 1970 for use in human arteries to stem aneurisms [11]. A variety of researchers had investigated the use of ferrofluid pumps and microvalves [[12], [13], [14], [15], [16], [17], [18], [19], [20], [21], [22], [23], [24], [25], [26], [27], [28], [29], [30]] with most applications of ID (inner diameter) being less than 1 mm and all being less than 5 mm. These applications range from the biomedical uses in directing cancer treatments to the lab-on-chip devices. In addition, studies on the hydrostatic plug with larger tube diameters, which respectively investigated a two-stage magnetic fluid vacuum seal in systems with varying radial clearances [31] and evaluated the magnetic fluid seals in dynamic environments [32], collectively underscore the advancements in utilizing ferrofluid plugs for larger diameters, providing a foundation for further research and development in this area.

However, when reviewing the current engineering applications [33], their focus is only on the smaller sealing applications commonly backed by mechanical seals for longevity. To the best of our knowledge, there are no attempts to use ferrofluid seals in an *ad hoc* manner where adaptability is championed over longevity of the seal. We were motivated to develop a capability of isolating high-pressure pipelines such as the Deepwater horizon oil spill with the use of *ad hoc* ferrofluid valves, stemming leaks quickly before they develop into larger disasters. To do so, ferrofluid valves need demonstrating in lower pressure (i.e., 0–1 MPa) process systems before they can be advanced into the higher-pressure arena of oil pipelines (i.e., 1–10 MPa). There already exists a commercial desire for *ad hoc* valving, which is demonstrated by industrial use of pipe freezing [34] and pipeline plugging [35]. In addition, its various characteristics make ferrofluids more advantageous. For example, the ice-plugs used in pipe freezing can slip and cause major damage with solid ice chunks hitting downstream equipment whereas ferrofluids once de-magnetized act as a fluid and can flow past these items without causing problems.

This paper aims to investigate the potential for using ferrofluid valves at macroscale pipes of ID in a range of 6∼18 mm under pressures up to 1 MPa. This is tackled by comparing the theoretical prediction and magnetic finite element analysis (FEA) against the hydrostatics experiments hoping to up-scale the results established for use at microscales and verify if they remain accurate when applied to larger systems. Since the creation of the theories [12], inconsistencies with the predictions were found when compared to the experimental data [19]. As such, to establish a sound basis for this investigation, magnetic FEA and physical testing have been pursued in the present study. These results are used to display the challenges involved with macroscale ferrofluid valves, and then recommendations are given for what areas need to be progressed to overcome these obstacles. Having much experience in a variety of process plants from dairy and alcohol, to pharmaceuticals, hydrogen and tritium, the reduction in valves would be not only a huge cost saving but also a reduced maintenance cost. The paper is organised as follows. Theoretical prediction of the burst pressure is introduced in Sec. 2. The selection of materials and various investigations are illustrated in Sec. 3. Results and discussion are presented in Sec. 4. Conclusions are drawn in Sec. 5.

## 2 Theory

**Fig. 1:**
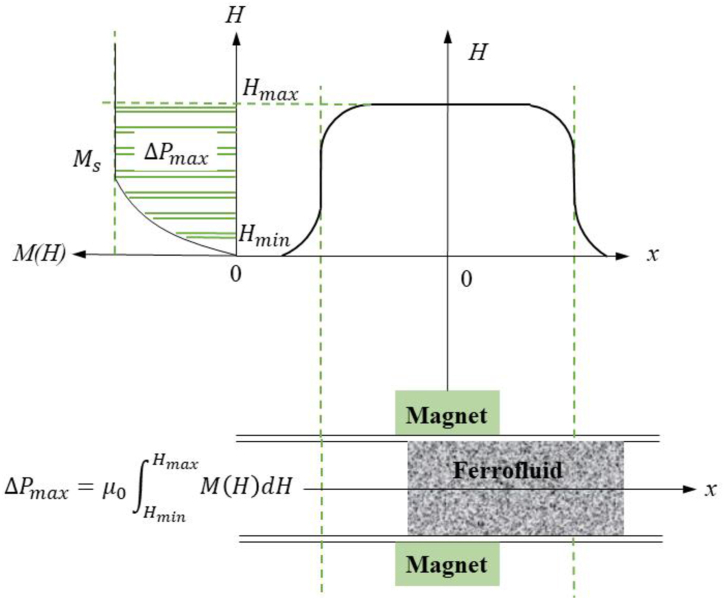
Schematic diagrams of the magnetic flux density distribution and magnetization curve (top panel), and a two-dimensional (2D) ferrofluid plug (bottom panel). Reproduced from Ref. [15].

Although many patents exist for the commercial application of ferrofluid seals, particularly in shaft seals, the mathematical and technical aspects have been widely researched and reported. To achieve the high burst pressure, a combination of fluids with high magnetic saturation, a magnetic field with high flux density, and a minimal gap between the magnets, are required [36]. A modified Bernoulli equation, which was derived by Rosensweig [37], is used to calculate the burst pressure of a static shaft seal:

(1) $$p_{1}+\rho gh_{1}-\mu_{0}\int_{0}^{H_{1}}M\left(H\right)dH=p_{2}+\rho gh_{2}-\mu_{0}\int_{0}^{H_{2}}M\left(H\right)dH$$

where $p_{1}$ and $p_{2}$ are the pressures of atmosphere on both sides of ferrofluid, ρ is the density of water, g is the gravity acceleration, and $h_{1}$ and $h_{2}$ are the hydrostatic heights of water. With the condition of $p_{1}{=p}_{2}$, the pressure difference $\Delta P=\rho g\left(h_{2}-h_{1}\right)$ to represent the burst pressure can be rearranged below [12,13,15]:

(2) $$\Delta P=\mu_{0}\int_{H_{1}}^{H_{2}}M\left(H\right)dH$$

where *M*(*H*) is the intensity of magnetization, *μ*_0_ is the permeability of free space, and *H*_1_ and *H*_2_ are the low and high magnetic field strengths at ferrofluid boundaries, respectively. Note that the magnetic field strength differs between the high-pressure and low-pressure fluid faces in Eq. (2), i.e., *H*_1_ < *H*_2_. Integrating Eq. (2) (i.e., the hatched area under the curve of saturation magnetization) returns the pressure difference across a single stage seal [37], demonstrating the effect of varying magnetic saturation across the two ferrofluid and retaining fluid interfaces (see Fig. 1).

When calculating the burst pressure, for the sake of simplicity, *H*_1_ can be taken as zero (i.e., the minimum value of magnetic field strength, $H_{\min}$) and *H*_2_ as the maximum strength (i.e., $H_{\max}$) between the magnets. In other words, the maximum loading condition occurs when the lower surface is in the region of almost zero magnetic field, and meanwhile, the upper surface is in the maximum field region. Assuming an unchanged *M*(*H*) across *H*_1_ and *H*_2_, which can be represented by the saturated magnetization of ferrofluid, *M*_s_, Eq. (2) is rewritten as follows [19,36]:

(3) $$\Delta P_{\max}=\mu_{0}M_{s}H_{max}$$

Eq. (3) can be employed to find the maximum static loading between a ferrofluid shaft seal and a ferrofluid valve. This had been proven experimentally by Perry and Jones [12], reporting a strong correlation between the theoretical values and experimental data. Although these developments are strong basis for the ferrofluid research, its level of correlation is not supported by solid practices, which eliminates the ability to establish the exact accuracy when the theoretical values are compared experimental data. It is also noted that although the deviation at this stage is lacking, its insight obtained in Eq. (3) makes our efforts worthwhile.

## 3 Materials and methods

### 3.1 Materials and pipe isolation

The composition of each item will vary, and it depends on the materials used and the ferrofluids intended purpose. For use with higher pressure differences, the nanoparticle quantities or size can be increased. However, this increases the overall viscosity of ferrofluids, which may not suit certain applications. One of ferrofluid producers Ferrotec provides key points such as the maximum Gauss (susceptibility to the magnetic field) and Centipoise (viscosity) of the ferrofluids but gives vast ranges for the constituent parts. For example, the fluid (EFH1) used in this study has nanoparticles (3%–15 %), surfactant (6%–30 %), and carrier fluid (55%–91 %) [38]. Although it is understandable that the wide range of composition may be intentional for protecting business interests, it reduces the ability to make scientifically accurate predictions. A typical composition of the ferrofluid used in the present experiments is listed in Table 1, and its influence is discussed later.

**Table 1:** A typical composition of the ferrofluid (EFH1) used in the present experiments.

Item	Percentage	Purpose	Typical materials
Nanoparticles	5 %	Magnetic acting part of fluid	Fe_3_O_4_, -Fe_2_O_3_ (Iron oxide)
Surfactant	15 %	Connects particles to the carrier fluid and repels particles from each other	Oleic acid, Citric acid, Soy lecithin
Carrier fluid	80 %	Fluid to suspend particles	Organic solvent, water, fluorocarbons, hydrocarbon oil

Although maintaining ferrofluid seals is not a new or solved phenomenon, it has been widely studied [4], i.e., tests in practical applications [39] and developments made towards increasing longevity with replenishment systems [5]. In these cases, longevity is the key parameter where a seal holding for six days is a failure. In an emergency isolation scenario, maintaining a seal long enough to create alterative long-term arrangements is key, and mere hours may be needed to weld in a mechanical valve arrangement or blanking flange. Perry and Jones also presented different modes of failure for ferrofluid plugs in vertical tubes with explanations of the resealing abilities [12]. The ability for a ferrofluid plug to reseal could be an advantage in some applications. However, each time a failure channel is created the passing fluid removes a portion of ferrofluid with it. In the context of creating isolation points within pipework, any passing of the isolation is a failure. For a ferrofluid plug to be a successful isolation, it must resist the maximum pressure that will be exerted upon it without any failure, in addition to the desired duration. Zhang et al. [40] compared the relative sealing pressures of various ferrofluid and magnetorheological seals with different pressures in shaft seals, showing successful sealing over 25 min with the magnetorheological fluids. However, it must be noted that the pressures applied to the ferrofluid seals were slowly raised and not in variable, instantaneous or turbulent manner that would be seen in industrial applications. Although sealing duration is an important factor for the commercial application, the present work is concerned on aligning the theoretical and experimental maximum pressures without concerning the element of time duration.

**Fig. 2:**
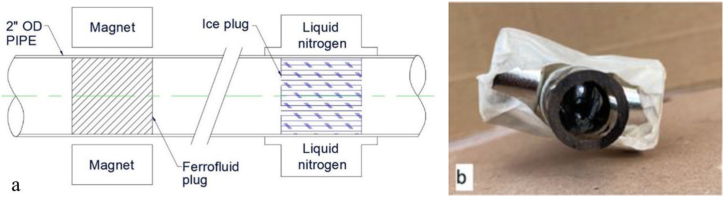
Two-dimensional (2D) schematic diagram comparing the similarities of a ferrofluid plug and an ice plug, both using an external resource (magnet or liquid nitrogen) to create blockage or isolation (a) and side view of a completed ferrofluid plug (b).

In this paper, we propose a novel isolation method to expand the application of ferrofluids to large scales. Fig. 2(a) and (b) illustrate a ferrofluid plug and an ice plug, respectively, both of which are operated by creating an internal isolation from an outside effect on the pipe. The ice plugs have been proven extensively in large diameter pipes [41] and are in successful commercial use, but the ferrofluid isolations have only been proven at the microscale in research applications. The extensive use of the ferrofluid plug for isolating pipelines shows a commercial desire to isolate pipe systems where positive isolations by permanent mechanical means are not possible. Pressure difference can be maintained by the ferrofluid plugs. However, its capacity at the macroscale (10–18 mm ID) have not been proven either theoretically or experimentally, which the present work will address.

### 3.2 Development experiments

In experimental measures, the null hypothesis theory was used. The null hypothesis is that the existing theoretical prediction for pressure drops across the ferrofluid plugs derived for the microscale will be accurate to within 5 % when applied to the proposed macroscale process systems. The alternate theory is that these calculations will not continue to remain accurate at the macroscale. To test this hypothesis, theory in Eq. (3) and FEA simulation were used to estimate the maximum burst pressures that can be held across a ferrofluid plug with varying magnet strengths and tube diameters, which was then compared against a series of physical hydrostatic experiments.

**Fig. 3:**
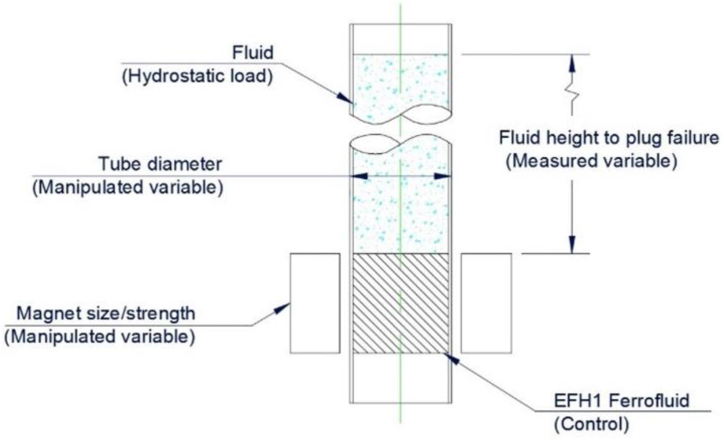
2D schematic diagram of hydrostatic experiment for comparison of theoretical and experimental values.

The main experiment in this paper is a hydrostatic loading, which is shown in Fig. 3, illustrating that a ferrofluid plug is held in position inside a tube via the external magnets. The tube is filled with a fluid, gradually increasing the hydrostatic loading on the ferrofluid plug. The approach has been applied to replicate experiments conducted by others at the microscale [12,42] and bring them to macroscale dimensions. As previously proven, when the loading pressure becomes too great for the ferrofluid plug, fluid will begin to pass with a failure channel allowing small quantities of fluid through before a complete failure of the plug. The ferrofluid (EFH1) is used throughout the control with the tube diameters and magnet strengths being manipulated variables. The measured variable is the hydrostatic height that the ferrofluid plug can hold, with comparisons taken from 12-off tube size and magnet combinations, which are shown in Table 2. BHmax 36–39 MGOe rated neodymium magnets (N38) were chosen due to their high magnetic strength, intended use at ambient temperature, ease of sourcing at various sizes and relatively low cost. Although higher strength magnets are available, they are more costly, reducing availability at smaller dimensions and becoming harder to physically handle. The magnet strengths sourced in our investigation were chosen due to their ease of handling and reduced risks of crushing. Due to the lack of research of ferrofluid plugs in pipe diameters greater than 5 mm ID, a range of larger tube sizes starting with 6 mm ID were selected, increasing in increments of 4 mm and all with 2 mm wall thicknesses.

**Table 2:** Manipulated variables such as magnet and tube sizes in hydrostatic experiment.

Magnet type	Magnet size	Tube size
Neodymium N38	N38 Ø19 mm OD x D3 mm N38 Ø20 mm OD x D10 mm N38 Ø25 mm OD x D25 mm	10 mm OD x 6 mm ID 14 mm OD x 10 mm ID 18 mm OD x 14 mm ID 22 mm OD x 18 mm ID

To establish a suitable experiment to compare with the theoretical prediction of burst pressures, four development experiments were undertaken to investigate both the various factors affecting the suitability of ferrofluids in process systems and the devise suitable parameters for the final hydrostatic experiment. The outcomes of four development experiments and the final experiment are detailed in Table 3. Surface tension (No. 3 in Table 3) significantly influences the stability and sealing capability of the ferrofluid plug within the pipe. High surface tension increases the cohesive forces among ferrofluid particles, enhancing the plug's integrity and its ability to resist pressure. A higher surface tension ferrofluid creates a more robust seal, which can improve the burst pressure, as it resists breaking apart under pressure [43]. On the other hand, the interaction between the ferrofluid and the tube material (No. 4 in Table 3) affects the wetting properties and adhesion of the ferrofluid to the pipe walls. A material with low surface energy (non-wetting) can decrease the adhesion, potentially leading to leakage paths and a reduction in burst pressure. Conversely, a material that allows better wetting and adhesion enhances the ferrofluid plug's stability and increases the burst pressure. In this study, acrylic tube was used. Acrylic has a smooth surface, which can improve the wetting properties and adhesion of ferrofluids. Proper surface treatment of acrylic can enhance these properties, resulting in better stability of the ferrofluid seal. The combination of high surface tension in the ferrofluid and a tube material that supports good adhesion and wetting properties is crucial for optimizing the burst pressure. Adjusting these factors can lead to better performance of ferrofluid isolation valves, especially in high-pressure environments.

**Table 3:** Summary of experimentation undertaken.

No.	Type	Description
1	Pilot study	Controlling ferrofluid plugs in PVC tubes
2	Immiscible fluids	Suitability and mixing of ferrofluids with other fluids
3	Surface treatments	Effects of surface treatments against ferrofluid staining
4	Tube materials	Investigation of ferrofluid staining on acrylic tubes
5	Hydrostatic load	Comparison between theoretical predictions and experimental data

**Fig. 4:**
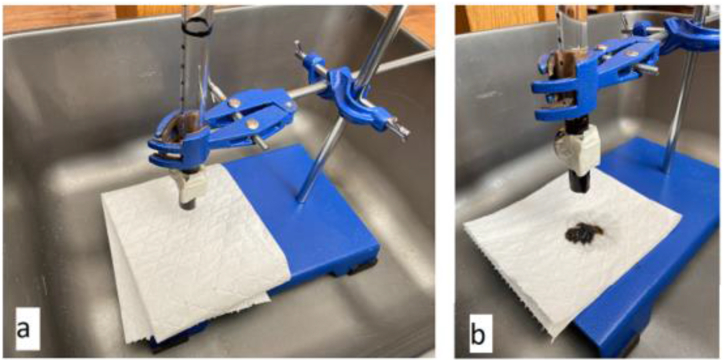
Hydrostatic experiment setup with an acrylic tube in a retort stand using tissue to highlight staining when the pug passes: (a) an unloaded plug and (b) a failed plug with stained tissue.

A hydrostatic test that consists of filling a vessel or piping system uses the weight/pressure of the water to find the failures. The experimental schematic shown in Fig. 4 is comparable to the experiment setup by Perry and Jones [12]. Fig. 4(a) shows that an acrylic tube clamped vertically in a retort stand with two magnets positioned at the opening with tape. A pipette was used to insert the ferrofluid inside the tube between the two magnets creating a ferrofluid plug. Water was then added to the opposite open end of the tube through a pipette, with a clean white tissue positioned under the tube. So, any water leakage is noticeable, and it has been shown in Fig. 4(b). Initial failure is exposed by a small leakage, and catastrophic failure is alerted by the entire contents of tube being released.

### 3.3 Theory-based hydrostatics

To theoretically predict the burst pressure for the hydrostatic loading, a four-step procedure is adopted.

Step 1: Establish the magnetic flux density in each of 12-off magnet/tube combinations.
Step 2: Calculate the magnetic susceptibility of EFH1 ferrofluid in each combination.
Step 3: Calculate the burst pressure that a ferrofluid plug maintains using Eq. (3).
Step 4: Convert the burst pressure to a hydrostatic loading.

**Fig. 5:**
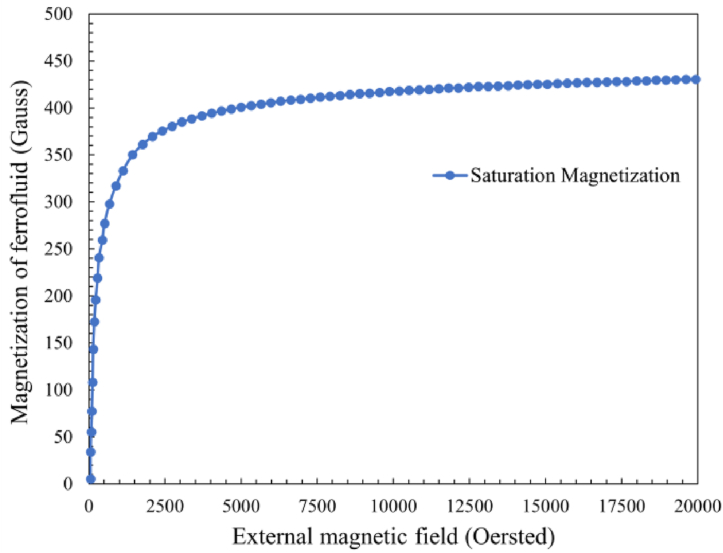
Saturation magnetization curve for EFH1 ferrofluid, provided by Ferrotec [38].

To increase the flux density within the tube and potential for holding pressure, a magnet was used either side of the tube in the experiment [15]. The calculated values of the magnet strength for each combination are shown in Table 4, representing the combined flux density at the central point of each tube, as found previously [12], which is the point of failure. To achieve the highest burst pressure, the ferrofluid needs to be magnetized as high as possible. Without magnetization, the nanoparticles within the ferrofluid will not align into chains and hold pressure. The magnetization curve for EFH1 ferrofluid is shown in Fig. 5, indicating the maximum magnetization of 0.044 T, which is achieved from an externally applied magnetic field of 1432.4 kA/m. To calculate the magnetization, the *H*-field strength of the magnet combinations is needed and can be converted from *B*-field strength in the first step, where $B=\mu_{0}H$. The values of *H*-field strength are shown in Table 5. As can also be seen in Fig. 5, a magnetic strength of 1432.4 kA/m is required to magnetically saturate EFH1 ferrofluid, at which the centre of any of the 12-off test combinations will not be achieved. As a result, the maximum magnetization of 0.044T will not be achieved, meaning that the full sealing potential of EFH1 ferrofluid will not be realised. The achievable magnetization for each combination is given in Table 5. Based on Eq. (3), the predicted burst pressures can be obtained and are given in Table 6, with values of magnetic field strength *H* and intensity of magnetization *M* taken from Table 5.

**Table 4:** Combined magnetic *B*-field strength at the central point of each magnet/tube combination in hydrostatic experiments.

Tube Magnet	Ø10 mm x D2 mm	Ø14 mm x D2 mm	Ø18 mm x D2 mm	Ø22 mm x D2 mm
N38 Ø19 mm x D3 mm	0.222 T	0.164 T	0.12 T	0.088 T
N38 Ø20 mm x D10 mm	0.48 T	0.36 T	0.269 T	0.203 T
N38 Ø25 mm x D25 mm	0.688 T	0.552 T	0.442 T	0.354 T

**Table 5:** Values of applied magnetic field intensity *H* and saturation magnetization *M*_s_ (within parentheses).

Tube Magnet	Ø10 mm x D2 mm	Ø14 mm x D2 mm	Ø18 mm x D2 mm	Ø22 mm x D2 mm
N38 Ø19 mm x D3 mm	176.9 kA/m (0.036 T)	130.8 kA/m (0.035 T)	95.5 kA/m (0.034 T)	70.1 kA/m (0.031 T)
N38 Ø20 mm x D10 mm	381.7 kA/m (0.04 T)	286.1 kA/m (0.0389 T)	214.22 kA/m (0.037 T)	161.6 kA/m (0.036 T)
N38 Ø25 mm x D25 mm	547.6 kA/m (0.041 T)	439.5 kA/m (0.04 T)	351.7 kA/m (0.04 T)	281.9 kA/m (0.039 T)

**Table 6:** Values of theoretically predicted burst pressure for each combination.

Tube Magnet	Ø10 mm x D2 mm	Ø14 mm x D2 mm	Ø18 mm x D2 mm	Ø22 mm x D2 mm
N38 Ø19 mm x D3 mm	6.368 kPa	4.578 kPa	3.247 kPa	2.173 kPa
N38 Ø20 mm x D10 mm	15.268 kPa	11.129 kPa	7.926 kPa	5.818 kPa
N38 Ø25 mm x D25 mm	22.452 kPa	17.58 kPa	14.068 kPa	10.994 kPa

### 3.4 FEA-based hydrostatics

To validate the theoretical predictions of magnetic field strengths between two magnets, magnetization finite element analysis (FEA) was performed. The complexity of magnetic field lines often limits the theoretical calculations to determining the strengths solely at the central point between the magnets [44]. However, FEA can measure the magnetic strength from any point within the magnetic field. For this purpose, a three-dimensional (3D) magnetostatic model was developed, consisting of a pair of N38 magnets, tubes of varying sizes, and a computational domain. A Magnetostatic module in ANSYS 2020 R1 was utilised for the FEA in the present work. The boundary condition of parallel magnetic flux was applied to the surfaces of the domain, ensuring that the magnetic flux passing through these surfaces is zero when it is parallel to the surface. In addition, data presented in Table 4 were used as the initial values in simulations, specifically for the magnetic field intensity and magnetization parameters. By enforcing this condition, the physical behaviour of the system is accurately represented, aligning the magnetic field with the configuration of the parallel magnets and the enclosure.

**Fig. 6:**
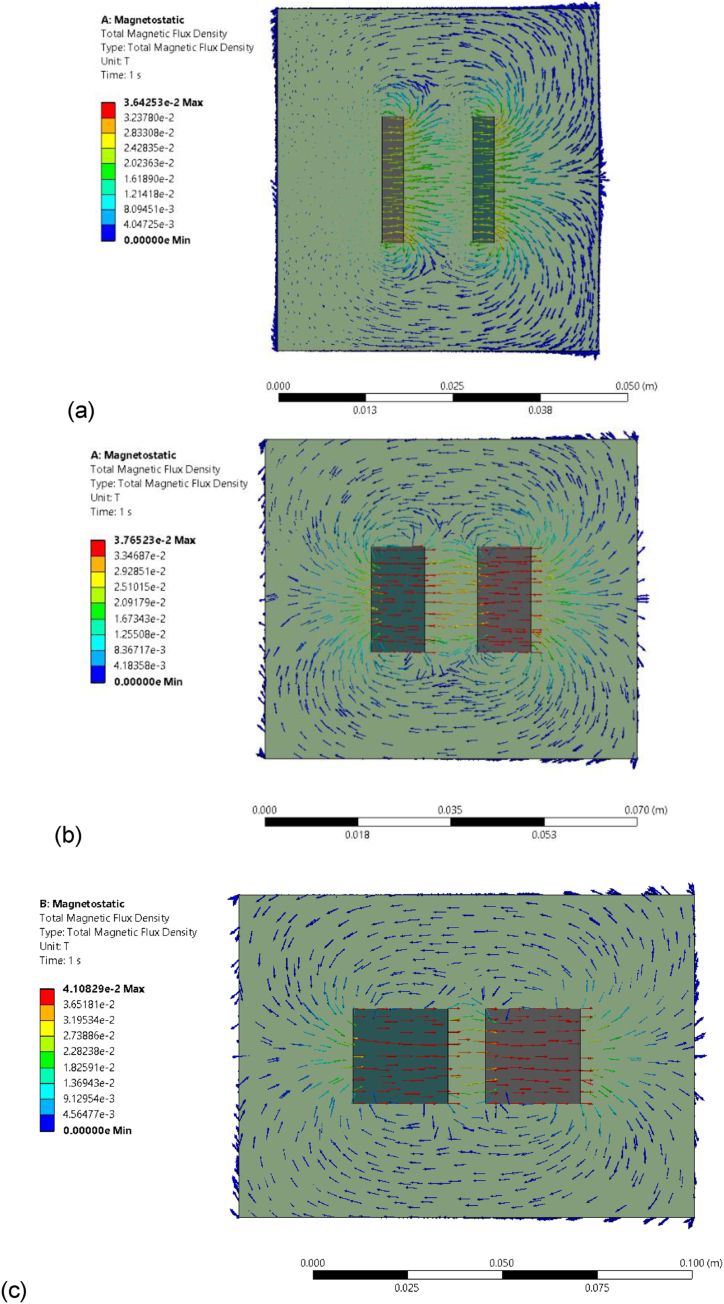
FEA results of the combined magnetic flux density between two N38 Ø19 mm x D3 mm (a), N38 Ø20 mm x D10 mm (b), and N38 Ø25 mm x D25 mm (c) magnets which are respectively distanced by a tube of Ø10 mm x D2 mm.

The mesh independency of FEA results was firstly evaluated by comparing the total magnetic flux density for different tube diameters (i.e., 10 mm, 14 mm, 18 mm, and 22 mm) at different mesh resolutions, i.e., from coarse to finer meshes. Fig. 6(a–c) showcase the total magnetic flux density for three different magnet diameters of Ø20 mm x D10 mm, Ø19 mm x D3 mm and Ø25 mm x D25 mm, which are respectively distanced by a tube of Ø10 mm x D2 mm. The results visually represent the distribution of magnetic flux density throughout the magnetic field. It is worth noting that the size of magnets can impact the distribution of the magnetic field. Larger magnets have a greater influence over a larger area, leading to a more uniform and well-distributed magnetic field (see Fig. 6(c)). In contrast, smaller magnets may exhibit more localised and uneven magnetic field distributions (see Fig. 6(a)). This difference in field distribution can affect the behaviour of the magnets and their interaction with the ferrofluids. Therefore, it is reasonable to expect that the size of magnets can impact the magnetic field distribution, which in turn can influence the results of burst pressure obtained in FEA simulations.

**Fig. 7:**
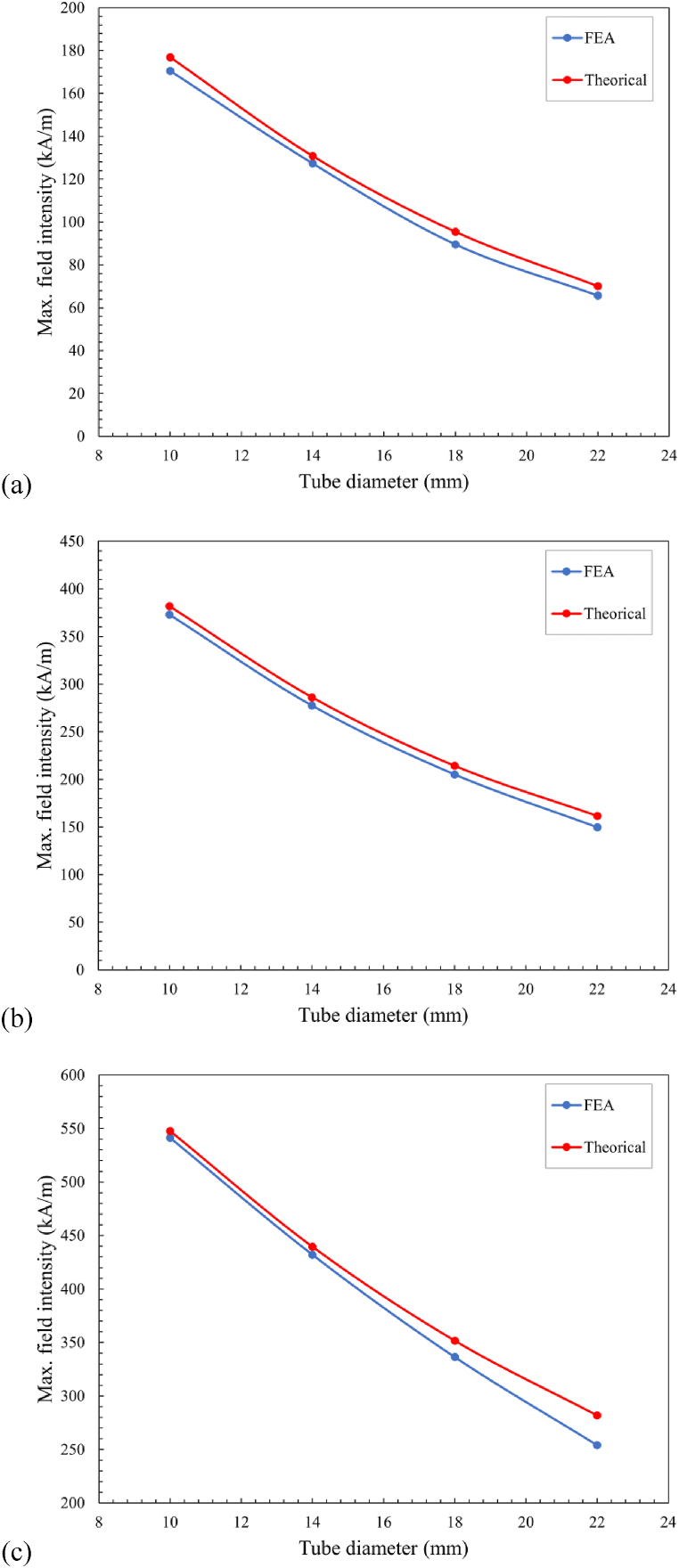
Comparison of the combined magnetic flux density between two: (a) N38 Ø19 mm x D3 mm; (b) N38 Ø20 mm x D10 mm; and (c) N38 Ø25 mm x D25 mm magnets from theory and FEA.

To further evaluate their accuracy, a comparison of maximum field intensity between FEA and theory for N38 magnets of different diameters has been made in Fig. 7. It shows a discrepancy between the theoretical results and the FEA results for different magnet sizes. Therefore, it is feasible that increasing the size of magnets can lead to a more efficient utilisation of the material's magnetic properties, resulting in an improved alignment with theoretical predictions. By aligning the FEA results with the theoretical calculations, this comparison validates the accuracy of the theoretical models and demonstrates their reliability in predicting the maximum field intensity. Based on this magnetostatic model, it enables us to measure the magnetic strength at any point within the magnetic field, overcoming the limitations of theoretical calculations that are restricted to the central point between the magnets. Table 7 presents the burst pressures, where the maximum magnetic flux density takes the value obtained in FEA.

**Table 7:** FEA-based burst pressure for each combination.

Magnet Tube	Ø10 mm x D2 mm	Ø14 mm x D2 mm	Ø18 mm x D2 mm	Ø22 mm x D2 mm
N38 Ø19 mm x D3 mm	6.138 kPa	4.455 kPa	3.046 kPa	2.036 kPa
N38 Ø20 mm x D10 mm	14.916 kPa	10.541 kPa	7.588 kPa	5.389 kPa
N38 Ø25 mm x D25 mm	22.191 kPa	17.28 kPa	13.456 kPa	9.911 kPa

## 4 Results and discussion

Table 8 summarises the values of burst pressure obtained in theory, FEA, and experiment, respectively. The burst pressures that are derived from the FEA simulated magnetic strengths reach greater agreement with the theoretical values with the increasing of magnet diameter. This suggests that magnetic saturation and magnetic field distribution are indeed influenced by the size of magnets without changing the EFH1 properties. As the size of the magnet increases, the magnetic domains within the EFH1 have more space to align with the external magnetic field, allowing it to reach its saturation point more effectively. Therefore, it is reasonable to expect that the size of magnets can impact the magnetic field distribution, which in turn can influence the results obtained from FEA simulations. As can be seen in every instance, both theoretical and FEA results are larger than the experimental data at smaller magnets (i.e., weak magnetic strength) and in larger tubes (i.e., at macroscale) but reach reasonable agreement at larger magnets (i.e., strong magnetic strength) in smaller tubes (i.e., at microscale). It is noted that repeating experiments will yield a larger dataset and increase the accuracy and reliability. To effectively analyse the experimental data, two statistical tools are used: the least squared linear regression and statistical inference using the *P*-Value. Use of the least square linear regression can increase the accuracy of results and compare the predicted burst pressures against the experimental ones, displaying the variance and correlation coefficient for each dataset. As already stated, the null hypothesis theory is used with the null remaining true unless a *P*-value of 0.05 is found. The *P*-Value can be obtained by comparing the predicted values and FEA results against the experimental data, assessing whether the probability of gaining the experimental measurement validates the alternate hypothesis.

**Table 8:** Comparison of the burst pressures predicted by theory, simulated using FEA or measured in experiment.

Magnet	Tube diameter	Theory (kPa)	FEA (kPa)	Experiment (kPa)
N38 Ø19 mm x D3 mm	Ø22 mm x D2 mm	2.173	2.036	0.687
	Ø18 mm x D2 mm	3.247	3.046	1.079
	Ø14 mm x D2 mm	4.578	4.455	1.961
	Ø10 mm x D2 mm	6.368	6.138	3.138

N38 Ø20 mm x D10 mm	Ø22 mm x D2 mm	5.818	5.389	2.55
	Ø18 mm x D2 mm	7.926	7.588	3.236
	Ø14 mm x D2 mm	11.129	10.541	5.982
	Ø10 mm x D2 mm	15.268	14.916	8.532

N38 Ø25 mm x D25 mm	Ø22 mm x D2 mm	10.994	9.911	5.099
	Ø18 mm x D2 mm	14.068	13.456	6.767
	Ø14 mm x D2 mm	17.58	17.28	10.983
	Ø10 mm x D2 mm	22.452	22.191	12.847

**Fig. 8:**
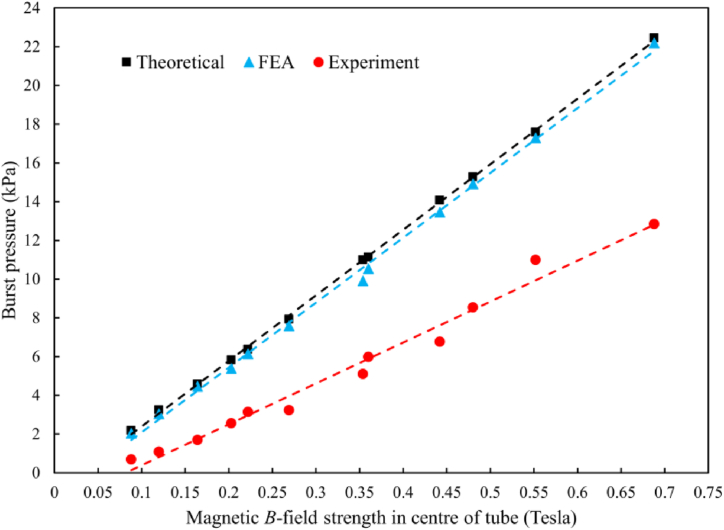
Least squared linear regression for the burst pressure in theory (black filled squares), FEA (blue filled triangles) and experiment (red filled circles) with the lines of fitting for each data.

The theoretical values, FEA results and experimental data are plotted in Fig. 8. It can be seen a strong linear relationship between the burst pressure and applied magnetic field regardless of the investigation approaches. The line of best fit for the theoretical prediction and FEA has a very high R2 correlation with the experimental measurement being less so but still strong: 0.9996 0.9976, and 0.9813, respectively. A strong correlation in both theory and simulation validates the calculations we have made in the terms of the maximum magnetic flux density (i.e., Eq. (3)). It is the same for the experimental results, indicating that each measurement was conducted in a similar manner without any rogue values or major inconsistencies. The line of fitting can be extended to approximate the required magnetic properties to gain the higher burst pressures at macroscales. This value might not be a complete reflection of accurate prediction in theory but reflects the potential development in experimental designs for a large-scale pipe isolation. It helps extend the current uses of ferrofluid microvalves, devising opportunities for application at a larger scale in process plant systems (ID ≥ 6 mm and 0–1 MPa). Taking the results from Fig. 8, the lines of fitting can be extended to approximate the applicable magnet strength to achieve a burst pressure of 1 MPa. However, the approximated magnetic strength needs to be used with caution and with an understanding that the potential inaccuracies in data will compound to a greater level when scaling up.

**Fig. 9:**
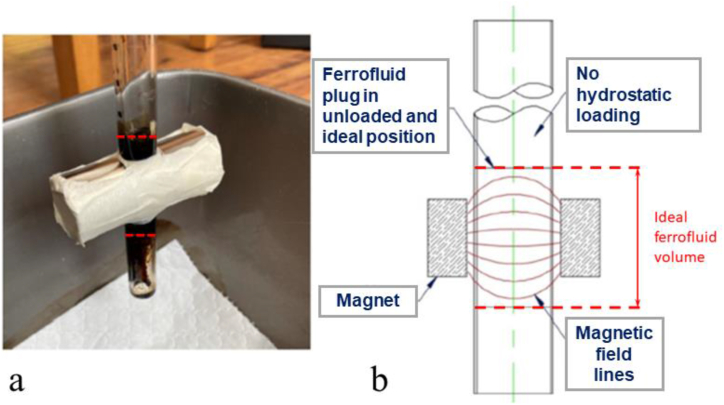
Ferrofluid plug positioned centrally between magnets with equal magnetization throughout the plug: (a) experiment setup and (b) schematic diagram. Red dashed lines indicate the boundaries of ferrofluids.

**Fig. 10:**
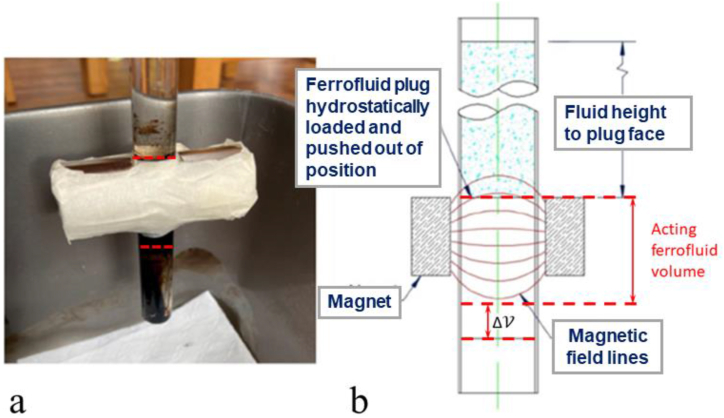
Ferrofluid plug hydrostatically loaded and pushed down out of a position of equal magnetization: (a) experiment setup and (b) schematic diagram. Red dashed lines indicate the boundaries of ferrofluids, showing that an amount of ferrofluid is pushed out of the magnetic field lines, represented by Δv.

During the development of experiments, it has been found that more attention needs to be paid when pairing a ferrofluid with the fluid which is resisting, in addition to the combination of ferrofluid and tubes materials. Both are fundamental points requiring further investigation for applying ferrofluids to the various fluids in process plants such as food, beverage, and pharmaceuticals, etc. The trends shown in Fig. 8 prove that using Eq. (3) with theoretical values and with FEA values generates correlation but gives low accuracy against the experimental data. A later study [19] showed a correlation between the theory and experiment, also reporting that the former overestimated the burst pressure up to 11 % higher than the latter, which aligns with the present findings. It is worth noting that lower accuracies are achieved with smaller magnet strengths and larger tube sizes amongst the data of burst pressure presented in Table 8. A postulation is that Eq. (3) does not account for the variance of reduced magnetic permeability through the acrylic tubing, instead of using a saturated value, which would weaken the impact of applied magnetic strength within the tube, creating a tighter performance envelope which is highlighted with the lower magnet strengths. This is demonstrated when unloaded plug is in equal magnetization (see Fig. 9 referring to ideal ferrofluid volume); when it is hydrostatically loaded, the plug is pushed out of the magnetic field lines (referring to acting ferrofluid volume in Fig. 10) until the high-pressure interface between the ferrofluid and water reaches the central point between the magnets (point of highest magnetic strength) before failing [19]. As can be seen in Fig. 10(a), the black ferrofluid plug has been pushed down between the magnets, and only the hydrostatic load can be seen in the tube above the magnets.

There are limitations that may have impacted the reliability of data found in this study. First, the range of composition varies in ferrofluids, and its compositions cannot be simply validated. The dataset collected from the experiments was small. However, the *R*-value showed high correlation between the data and the line of fitting, indicating the experimental results were in good agreement. The big challenge in this study is not being able to determine the actual experimental flux densities of magnetic inside the tubes, overcoming factors such as unknown the actual magnet strengths. Thus, Eq. (3) theoretically generates values, which correlate with the experimental results, but has lower accuracy. Furthermore, the deviation of both theoretical and FEA values of the burst pressure from the experimental data may arise from the assumptions made in Eq. (3). Recalling that Eq. (2) was induced to Eq. (3) by taking lower and upper limits $H_{1}$ and $H_{2}$ as their minimum and maximum values of $H_{\min}$ and $H_{\max}$, the maximum pressure predicted in Eq. (3) is not the hatched area under the curve of saturation magnetization (see Fig. 1) but an area of a rectangular shape with a length of $H_{\max}$ and a height of $M_{s}$. Especially, as shown in Fig. 10(b), an amount of ferrofluid is pushed out of the magnetic field lines, which is represented by Δv, and only the acting volume of ferrofluid and its displacement increases. It may be the reason why the experimentally measured values of burst pressure are lower than both results of theory and FEA.

## 5 Conclusions

The present work has investigated the current uses of ferrofluid microvalves and devised opportunities for application at a larger scale system (Ø ≥ 6 mm ID and *P*-value 0–1 MPa). The characteristics of ferrofluids are based on its three constituent parts: the carrier fluid, the surfactant, and the magnetic domain within the nanoparticles. The success of a ferrofluid plug for use as an isolation depends on its strength in resisting pressure, focusing on a product of the magnetic susceptibility of ferrofluids and the applied magnetic strength within the pipe. As shown in this study, the key factors affecting a ferrofluid plug to hold high pressures is to maximise the magnetic strength at the central point within the tube and across the interface between the ferrofluid and water as they move under the strain. It also discussed which parts of this three-part setup (magnetic strength, ferrofluid magnetic susceptibility, and tube sizes) need development to take these ferrofluid plugs close to the industrial application.

The existing theory used for predicting the burst pressure in microvalves has been extended to the macroscale, including the experimental development and FEA performance. Strong correlation was found between each dataset although on average the experimental values of the burst pressure were lower than both predicted and simulated ones. Based on these results, it was concluded that to achieve a burst pressure of 1 MPa with a similar experimental setup will require very high magnetic strengths. It was also found that Eq. (3) was successful in predicting the burst pressures within the same order of magnitude but the saturated magnetic permeability assumed in the theoretical predictions was responsible for the overestimated values against the physical experiments. Solution to increasing the pressure differences is to enlarge the magnetization at the central point of the tube by either increasing the magnetic strength in this region or increasing the magnetic susceptibility of the ferrofluid. The former focuses on the magnetic field by using pole shoes [45], and the latter suggests a blend of ferrofluids with the magnetorheological fluids [46].

Applying ferrofluids in the process engineering or oil/gas industries for use along with many other various fluids will face many challenges. This paper investigates whether *ad hoc* ferrofluid valves could be used in place of fixed mechanical valves, reducing the number of pipe-brakes and gaskets involved in a process system. Here, it focuses on the mechanical properties of ferrofluid plugs, with further study invited from others to access chemical aspects of specific industry needs. With the successful pursuit of the recommendations given, ferrofluid valves will hold a real-world application although they are currently only used in lower-pressure systems. Through development this can progress towards a vital step of ferrofluid plugs being able to perform in high-pressure environments and stem oil pipeline leakages in favour of a positive isolation.

## CRediT authorship contribution statement

**Jake O. Emmerson:** Writing – original draft, Visualization, Validation, Resources, Methodology, Investigation, Formal analysis, Data curation, Conceptualization. **Amirali Shateri:** Writing – review & editing, Visualization, Validation, Software, Methodology, Investigation, Formal analysis, Data curation. **Jianfei Xie:** Writing – review & editing, Validation, Supervision, Software, Resources, Project administration, Methodology, Investigation, Formal analysis, Data curation, Conceptualization.

## Declaration of competing interest

The authors declare that they have no known competing financial interests or personal relationships that could have appeared to influence the work reported in this paper.
