# Supplementary material for: Use of magnetic fluids in process system for pipe isolations
Source: Heliyon. 2024 Jul 26;10(15):e35221. doi: 10.1016/j.heliyon.2024.e35221 (PMC11333900; doi:10.1016/j.heliyon.2024.e35221)
Supplement: Multimedia component 1 [file mmc1.docx]

**Experimental measures and FEA practices**

**S.1 Tube diameters**

To validate the findings of previous studies at a larger scale, a variety of tube sizes from Ø10 to 22 mm were chosen for investigation. The tube sizes increase at intervals of 4 mm, with all having a 2 mm wall thickness. Each outer and inner dimension was measured with a vernier caliper (Fig. S1), with the results shown in Table S1. The strength of the combined magnetic force within the tube is highly dependent on the positioning of the magnets: the closer the magnets, the higher the combined flux density and a better ability to hold pressure.


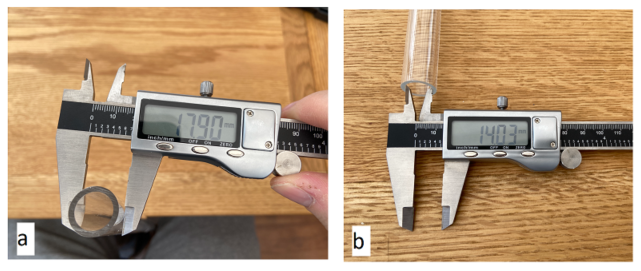


**Fig. S1.** Example of vernier measurement using Ø18 mm x 2 mm thick tube: a) OD; and b) ID.

The highest variance was found in ID of the Ø10 mm x 2 mm thick tube with -2.7% variance. When analyzing the experimental results, the variance from theoretical tube sizes to actual tube sizes will be considered. This is a critical importance when comparing the values.

**Table S1** Results of vernier caliper tube measurements showing discrepancies in OD and ID.

| Tubes | Ø10 mm x 2 mm | Ø14 mm x 2 mm | Ø18 mm x 2 mm | Ø22 mm x 2 mm |
| --- | --- | --- | --- | --- |
| Measured OD | 9.82 mm | 13.81 mm | 17.9 mm | 21.88 mm |
| OD Variance | -1.8% | -1.4% | -0.6% | -0.5% |
| Measured ID | 5.84 mm | 10.16 mm | 14.03 mm | 18.04 mm |
| ID Variance | -2.7% | +1.6% | +0.2% | +0.2% |

**S.2 Magnet dimensions and strengths**

All six magnets used in the experiment were also measured using a vernier caliper, although measured with a non-digital plastic version due to the magnet’s attraction to the metal digital calipers, which hindered the accurate measurements, as shown in Fig. S2.


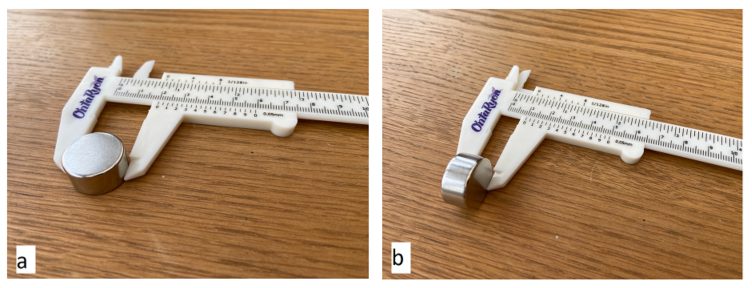


**Fig. S2.** Example of vernier measurement using Ø20 mm x 10 mm thick magnet.

The vernier measurements of the magnets displayed no discrepancies from the manufacturers claimed dimensions. To determine the B-field strength of the magnets a gauss-meter could be used, however, one could not be sourced in this paper due to the high cost of these units. As such the strength of each magnet has been determined theoretically, we take the assumption that the magnets have been magnetized to saturation and have not lost any strength via manufacturing defect or have suffered demagnetization.

**S.3 Positioning magnets**

For quick and secure fastening of the magnets to the tube, masking tape was used, as shown in Fig. S3. The bottom edge of the magnet was positioned 30 mm from the tube opening, with one magnet secured before the second with a separate piece of tape. Opposite magnetic poles are positioned facing one another, with alignment made via visual means.


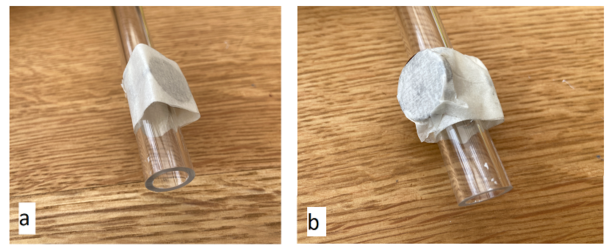


**Fig. S3.** Example of method used to position magnets: a) single magnet; and b) two magnets.

**S.4 Building the ferrofluid plug**

Ferrofluid was transferred using a pipette from a vial into the magnetic gap within the tube. As seen in Fig. S4.1(a), a small amount of ferrofluid has been placed inside the tube and has been magnetized against the tube wall. Fig. S4.1(b) illustrates a complete ferrofluid plug.


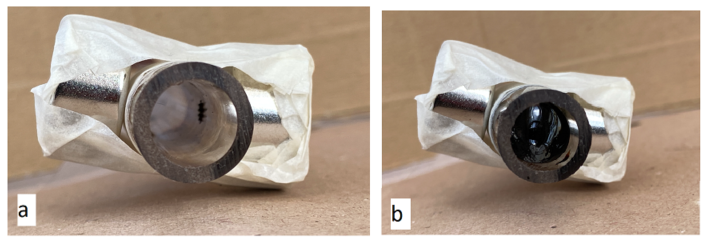


**Fig. S4.1** Ferrofluid insertion into the tube: a) small quantity magnetized to the inner wall; and b) completed ferrofluid plug.

When adding ferrofluid into the tube to create the ferrofluid plug, the quantity of ferrofluid was increased until ferrofluid began to run along the tube signaling that the plug had occupied the available magnetic field within the tube, as detailed by Perry and Jones [17]. The ferrofluid plug once completed extended out either side of each magnet, as shown in Fig. S4.2.


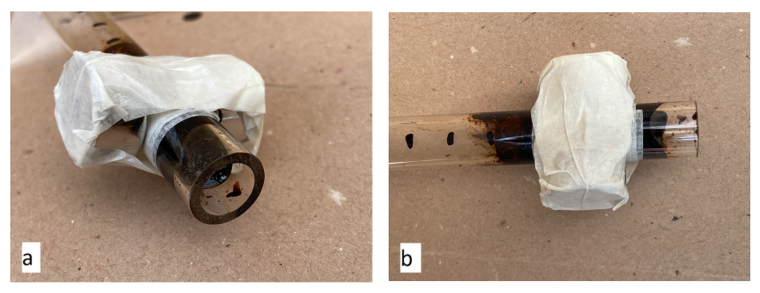


**Fig. S4.2** Completed ferrofluid plug (Ø20 mm x 10 mm thick magnet and Ø18 mm tube).

**S.5 Positioning tube in stand**

Once the plug was completed within the tube, it was carefully fastened in the retort stand, as shown in Fig. S5(a), with the tube opening positioned 50 mm from a piece of clean tissue. In each setup, the tubes vertical positioning was kept straight using a spirit level, as shown in Fig. S5(b). In some cases, excess ferrofluid dripped from the tube, this was wiped clean, and the tissue was replaced (see Fig. S5(c)).


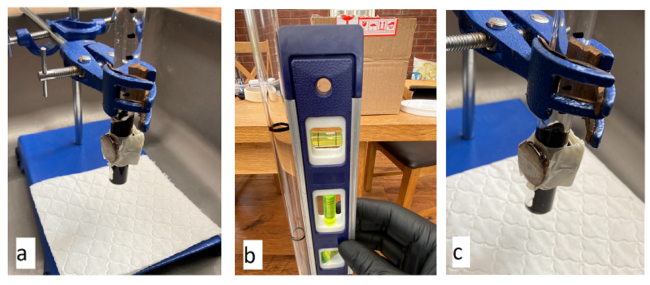


**Fig. S5.** Positioning of tube in retort stand: a) in clamp; b) straightening with spirit level; and c) excess cleaned.

**S.6 Development experiments**

**S.6.1 Pilot study**

The pilot study was conducted to investigate the ferrofluid plugs moving water through 5 mm ID PVC tubes. There are two aims in this pilot study: to move water through the system using a ferrofluid plug controlled via the external magnets; and to gain some firsthand experience handling ferrofluids. Two lengths of PVC tubing are connected to a valve block: one end extending 3 m into the air (Fig. S6.1(a)); and the other connected to a glass pot for introducing the ferrofluid (Fig. S6.1(b)).


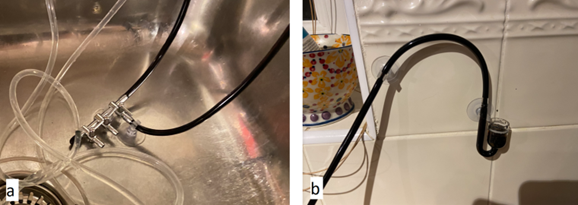


**Fig. S6.1** Pilot study experiment: (a) One end connected to the air; and (b) the other connected to a glass.

The valve block was opened to allow the system to be filled with water and remove any airlocks forming. Ferrofluid was added to the system via the glass pot and guided into position close to the valve block via the external magnets. Once sufficient ferrofluid had been added to the system, the valves connecting the two lines were opened with two external magnets being used to move the position of the ferrofluid plug. This movement of the ferrofluid plug corresponded to a clear movement in the water height in the opposite tube. The experiment was successful in achieving both aims. However, it was found that the tube material was stained and the water/ferrofluid interface mixed heavily, which made the visual identification of the ferrofluid plug incredibly difficult.

**S.6.2 Immiscible fluids**

The immiscible fluids experiment was built on the findings in the pilot study, looking to investigate fluids with a strong boundary with EFH1 ferrofluid that would not readily mix. EFH1 uses a hydrocarbon base, which is a nonpolar substance, so its immiscibility will be tested with the polar substances with varying concentrations of saltwater. The aim was to find a suitable immiscible fluid to use in the system alongside EFH1 ferrofluid for future experiments ensuring that the interface does not mix (this is not only important for the visibility but also for the performance of the ferrofluid plug). Four 50 ml glass test tubes were filled with varying quantities of salt with 0%, 10%, 19% and 29% from left to right, as shown in Fig. S6.2(a).


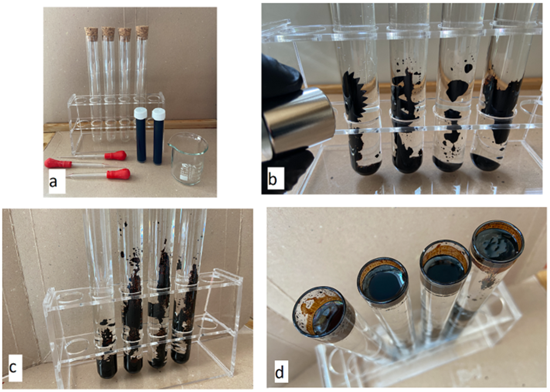


**Fig. S6.2** Immiscible fluids experiment: a) Equipment setup with tubes filled with varying concentrations of saltwater; b) ferrofluids added to the tubes and a magnet used to agitate the fluids; c) staining of ferrofluids on walls of tubes; and d) ferrofluid on surface of tubes.

When dropping ferrofluid onto the fluid surface, approximately 60% fell through the surface of the saltwater to the bottom of the test tube with ferrofluid falling quickest in a water-only tube and the slowest in the highest concentration of saltwater, as shown in Fig. S6.2(b). The ferrofluid in each test tube was agitated with ferrofluid moving up along the walls of the tube (see Fig. S6.2(c)). No noticeable difference was found in the immiscibility of the ferrofluid with any of the saltwater concentrations although the tubes with higher salt contents displayed more staining on the walls of the tubes and a higher quantity of ferrofluid that had risen to the surface (see Fig. S6.2(d)). It was expected that the water-only test tube would readily mix with the ferrofluid, as seen in the pilot study even this was not the case. The goal of finding an immiscible fluid to use for the next experiments has been achieved, and this will simply be with water only as no gain is made by using the saltwater.

**S.6.3 Surface treatments**

The surface treatment experiments were also built on the findings in the pilot study, i.e., ferrofluid heavily staining PVC tubes. Although it is not critical to investigating the hydrostatic loading on a ferrofluid plug, this staining will hinder our observations. The aim was to compare the hydrophobic coatings on PVC pipes to find a suitable pairing for the hydrostatic experiment. PVC tubing was cut into 100 mm long pieces with each end plugged with a cork bung. The hydrophobic coatings used in the treatment were Scotch guard water repellent (SG), Rain-x glass water repellent (XG), and Rain-x plastic water repellent (XP). Four test pieces were prepared, using one control tube filled with the water only and the others with the coating described above. Each coating was applied to the tubes and left to soak before being drained and filled with water. When ferrofluid was added to the water-only control tube, the ferrofluid formed an immiscible ball and fell to the bottom of the test tube. When it was agitated with a magnet up the sides of the pipe, the inner wall of the PVC tube stained heavily, as can be seen in Fig. 5. When ferrofluid was dropped into the other three test pieces with hydrophobic coatings, the ferrofluid dispersed and instantly mixed inside the tube and all three became a dark water mix.


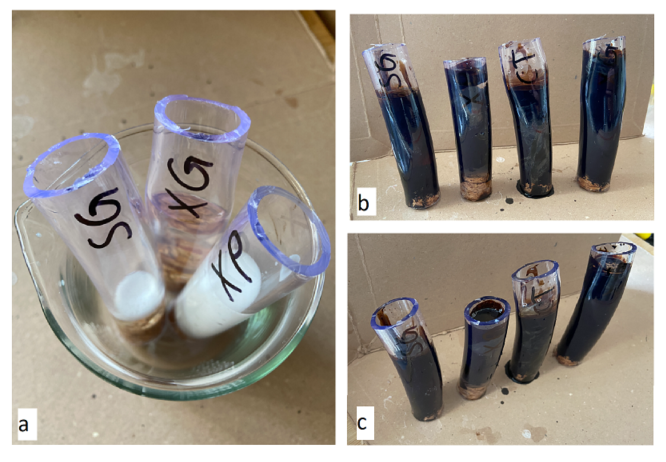


**Fig. S6.3** Surface treatments experiment: a) Test pieces with coatings being applied; b) and c) show staining of the PVC in all test pieces (left to right: SG, XG, water-only control, and XP).

The water-only control tube gave the same result as found in the first two experiments: the ferrofluid formed a self-contained immiscible ball in water. However, the PVC piping stained much more heavily than the borosilicate glass in the second experiment. An oversight in this experiment design was that the hydrophobic coating would only affect the surface of the pipe, as opposed to being mixed within the entire water sample of the test piece. When ferrofluid was introduced to these test pieces, the hydrophobic properties had an adverse effect and split the ferrofluid apart. Due to the adverse staining found in each test piece, we need to find a new pipe material for our hydrostatic experiment.

**S.6.4 Tube materials**

The tube materials experiment was then conducted to investigate the potential use of the acrylic tubing. The aim was to test the effects of EFH1 ferrofluid staining on the acrylic tubing, comparing water against the saltwater. Two pieces of acrylic tubing were cut into 100 mm long pieces and cork bungs were used to plug the bottom open ends. Both tubes were fixed into a retort stand, one filled with water and the other with a 17% saltwater. 10 ml of ferrofluid was then dropped into each tube, both forming an immiscible ball and falling to the bottom of the test tube, although over time some of the ferrofluid rose to the surface of the saltwater tube. A magnet was used to agitate the ferrofluid in each test tube, with both samples showing staining of the acrylic tube wall, as can be seen in Fig. 6.


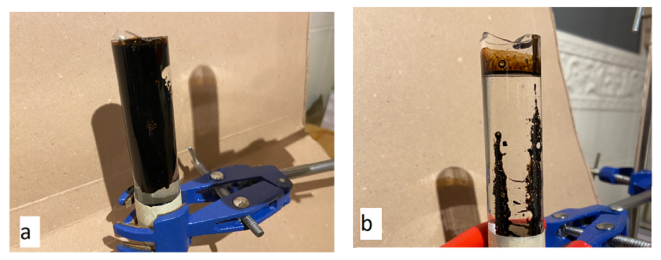


**Fig. S6.4** Tube materials experiment: a) Acrylic tube with the saltwater and b) acrylic tube with water.

The saltwater solution generated adverse effects not desirable for the hydrostatic loading experiment, namely the ferrofluid rising to the surface and heavy staining on the internal walls. The water-only test yielded more desirable characteristics. The key purpose of the hydrostatic experiment is to test the capable loading of a ferrofluid plug, and the tube staining characteristics are not of high importance. However, they hinder quality observations inside the tubes. It has therefore been decided to proceed with the acrylic tubing and water for the final experimentation.

**S.7 Filling water and viewing water loss**

The experimental measurement and theoretical prediction are based on a hydrostatic principle, such that the loading of the plug should be gradual as to not introduce a dynamic loading. A pipette was used to draw water from a glass beaker, with the water expelled from the pipette into the upper open end of the tube against the inside wall of the tube to reduce any dynamic loading conditions.

To measure the fluid height in the tube, each tube was marked with increments of 10 mm and each 100 mm marked by a complete circular band around the tube. To recognize the failing of the ferrofluid plug, a piece of white tissue was placed underneath the tube, as shown in Fig. S7. When a slight drip passes through the ferrofluid plug, drops of ferrofluid can be clearly seen on the tissue. In the scenario of a catastrophic failure, the entire contents of the tube empties, which is quite evident and is recorded as the failed value in our investigation.


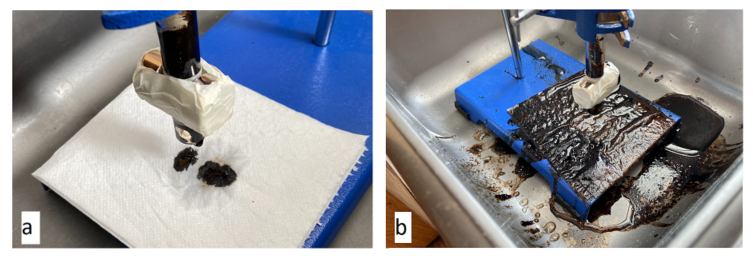


**Fig. S7** Example of ferrofluid plug failure: a) small passing and b) catastrophic failure.

**S.8 FEA practice of magnetic field strength**

To validate the theoretical predictions of magnetic field strengths between two magnets, magnetization finite element analysis (FEA) was performed. For this purpose, a 3D magnetostatic model was developed, consisting of a pair of N38 magnets, tubes of varying sizes, and a computational domain (See Fig. S8.1)

In this study, the mesh independency of FEA results was evaluated by comparing the total magnetic flux density for different tube diameters (10 mm, 14 mm, 18 mm, and 22 mm) at three different mesh resolutions: coarse mesh, fine mesh, and finer mesh. The mesh resolution was increased from 5,000 to 45,000 elements. As the mesh was refined, the estimated magnetic flux density also increased, ranging from 24.241 mT for the coarse mesh to 38.559 mT for the finer mesh (see Fig. S8.2). However, the results obtained from 40,000-45,000 did not significantly affect the magnetic flux density. Therefore, it can be concluded that mesh independence was achieved in this study.

| 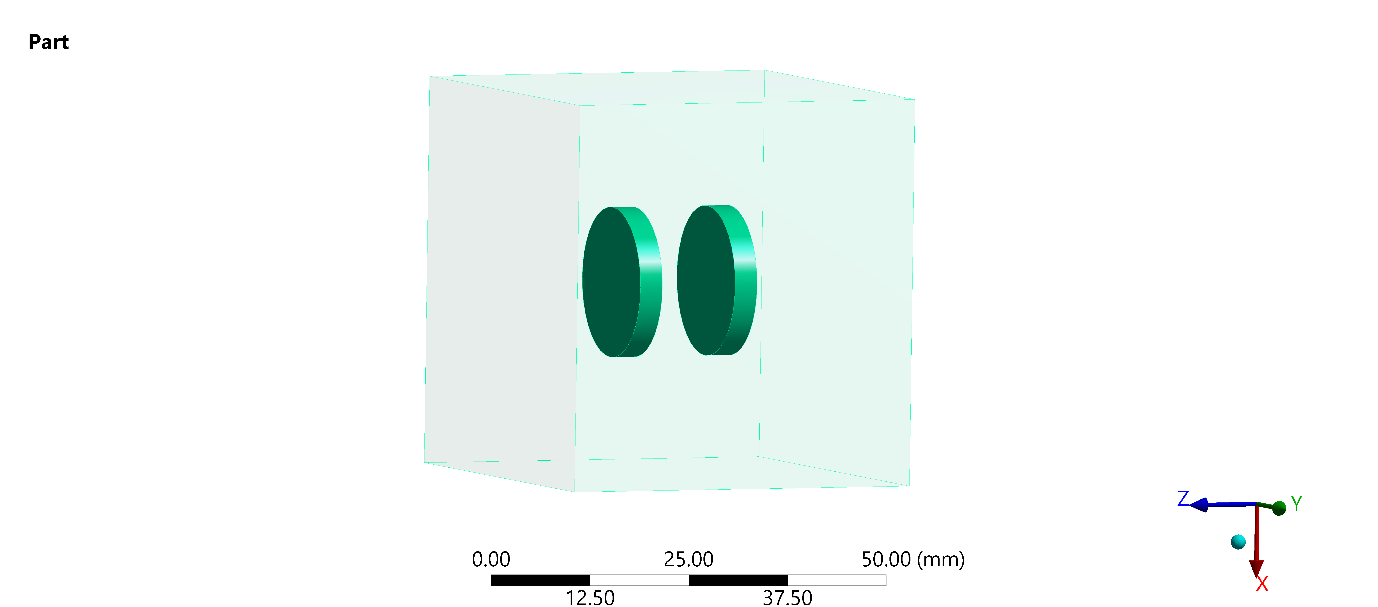 | 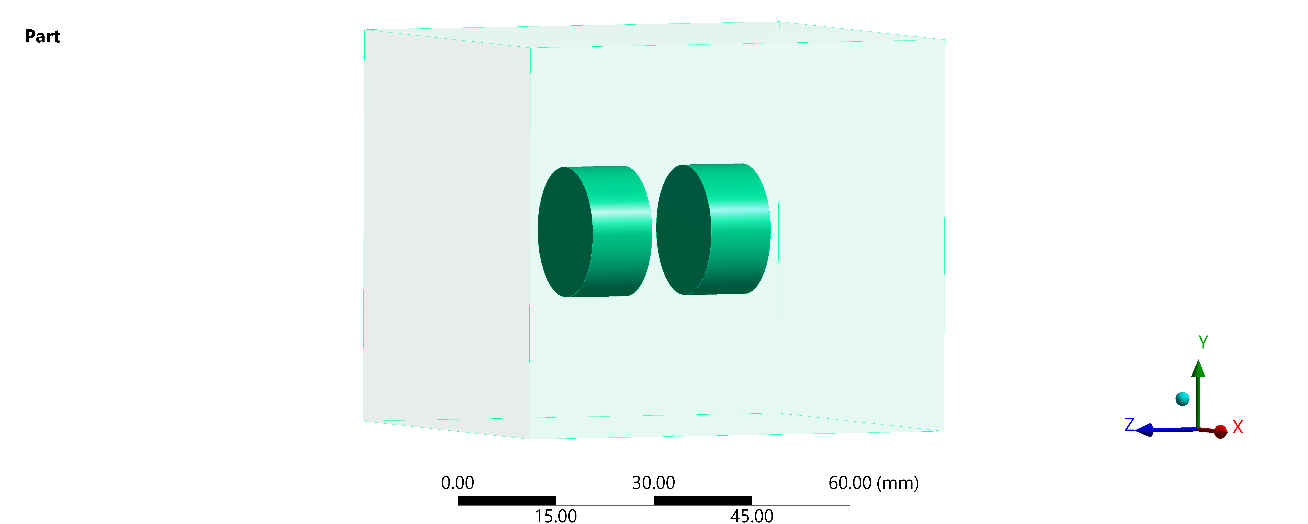 |
| --- | --- |
| (a) | (b) |
| 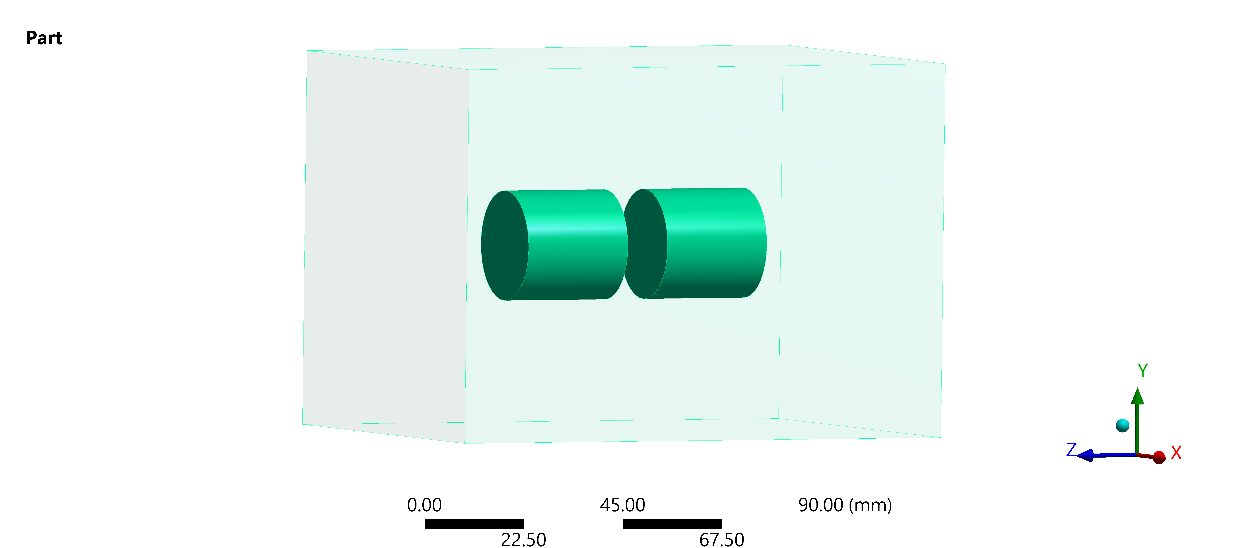 | 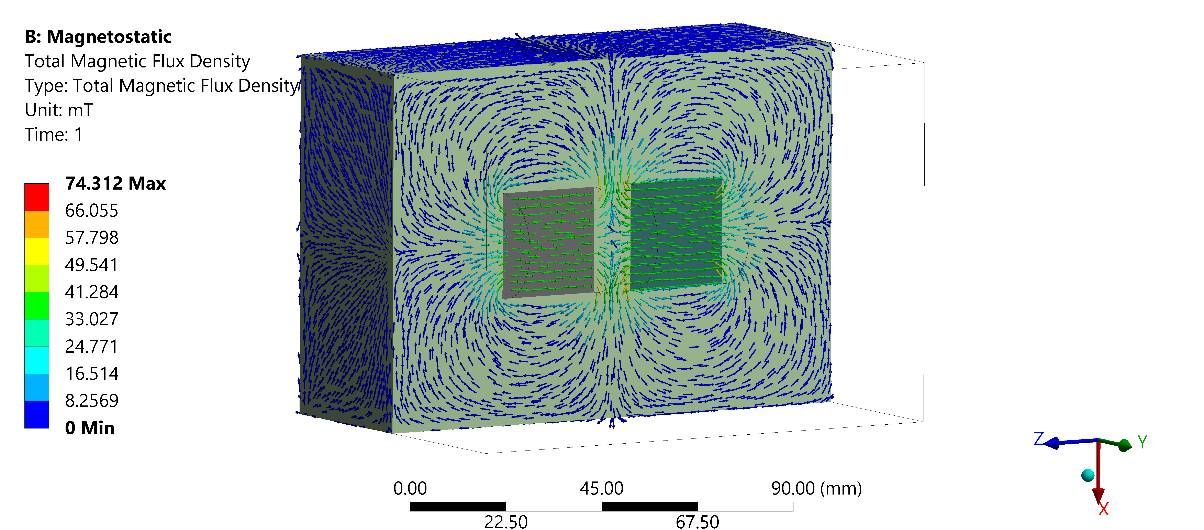 |
| (c) | (d) |

**Fig. S8.1** Three-dimensional (3D) geometry of magnets and computational domain; (a) Ø20 mm × D=10 mm and (b) Ø19 mm × D=3 mm, (c) Ø25 mm × 25 mm and (d) the section plane of computational domain for magnets Ø25 mm × 25 mm using a Ø10 mm × D=2 mm tube.


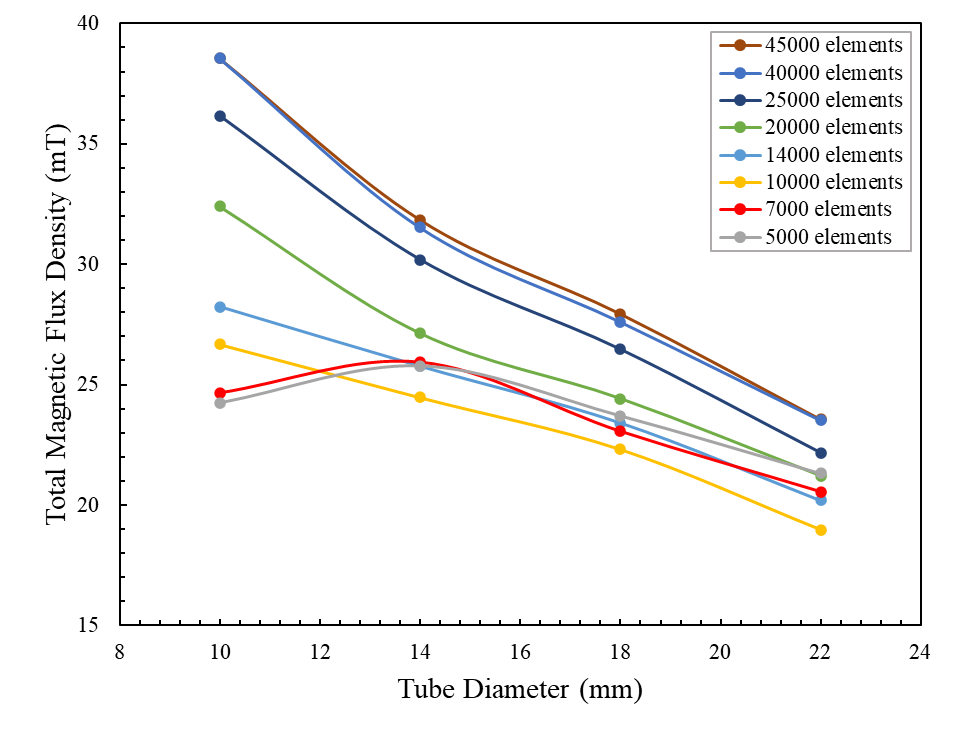


**Fig. S8.2** Mesh independence study: Comparison of maximum magnetic force (N) for N38 magnets Ø20 mm × D10 within different tube diameters (10, 14, 18, and 22 mm).
